# An Aberrant Right Colic Artery Arising From the Right Gastroepiploic Artery

**DOI:** 10.1111/ans.70787

**Published:** 2026-06-09

**Authors:** George Triantafyllou, Ioannis Paschopoulos, George Tsakotos, Orestis Lyros, Panagiotis Kokoropoulos, Maria Piagkou

**Affiliations:** ^1^ Department of Anatomy, School of Medicine, Faculty of Health Sciences National and Kapodistrian University of Athens Athens Greece; ^2^ Fourth Department of Surgery Attikon University Hospital, National and Kapodistrian University of Athens Athens Greece

1

During routine anatomy dissection of a 68‐year‐old male donated cadaver, an unexpected anatomical variant was identified. Dissection of the abdominal cavity was initiated with an incision along the linea alba. The stomach and large intestine were identified, along with the gastrocolic ligament that was dissected to expose the pancreas and the abdominal vessels. Then, the coeliac trunk (CeT) and its branches were identified by careful dissection. The gastroduodenal artery (GDA) was observed posteriorly to the first part of the duodenum, while it was typically bifurcated into the right gastroepiploic artery (RGEA) and the superior pancreaticoduodenal artery.

The RGEA, with a diameter of 5.4 mm at its origin, typically coursed towards the greater curvature of the stomach. Distally to its origin (length: 21.9 mm), it provided an arterial branch with a diameter of 3.7 mm. This branch coursed posterior to the transverse mesocolon and the mesentery of the proximal jejunum, laterally and caudally towards the hepatic flexure and the ascending colon, providing branches via the marginal artery (Figures 1 and 2). Nevertheless, there was no branch corresponding to the typical right colic artery (RCA). Therefore, the variant arterial branch corresponded to an aberrant RCA. Otherwise, the major abdominal arteries (CeT, superior and inferior mesenteric arteries) were free of variants.

**FIGURE 1:**
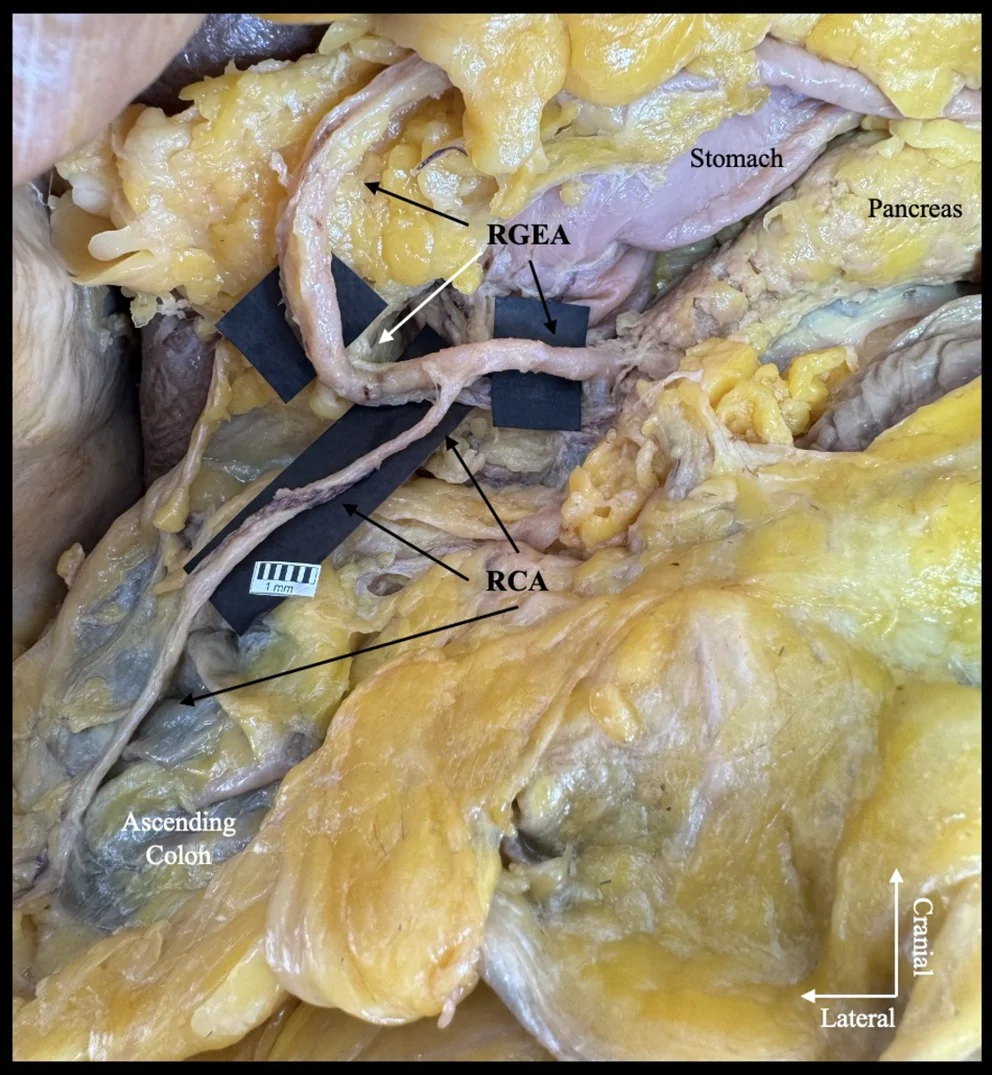
Cadaveric dissection of aberrant right colic artery (RCA) arising from the right gastroepiploic artery (RGEA).

**FIGURE 2:**
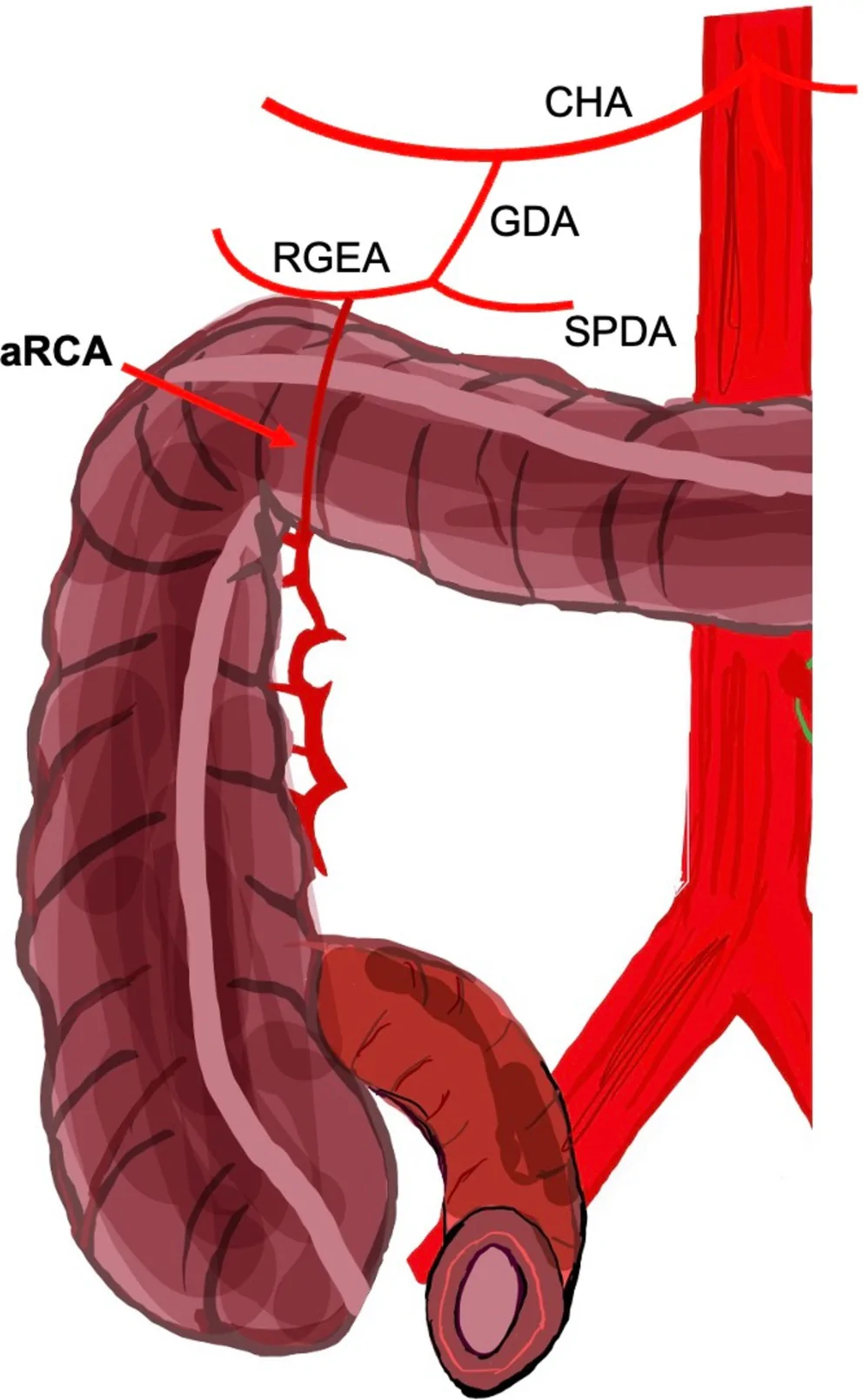
Schematic representation of aberrant right colic artery (aRCA) arising from the right gastroepiploic artery (RGEA). CHA, common hepatic artery, GDA, gastroduodenal artery, SPDA, superior pancreaticoduodenal artery.

We reviewed the literature to identify studies and cases reporting variable RCA origin patterns. Although *Bergman's Comprehensive Encyclopedia of Human Anatomic Variation* [1] and recent anatomical studies performed by Gamo et al., Haywood et al., and Wu et al. offered valuable anatomical details for the RCA, there was no identification of RGEA origin [2, 3, 4, 5]. The only report describing such origin was identified by Zoulamoglou et al. during total mesocolic excision. However, their intraoperative figures did not accurately depict this variation like the current cadaveric case [6].

The aberrant origin of the RCA from the RGEA is best explained by the persistence of primitive vascular channels formed during early fetal development. Initially, the dorsal abdominal aorta gives rise to a series of paired ventral splanchnic arteries that supply the primitive gut. The foregut, midgut, and hindgut are subsequently supplied by the three definitive, unpaired branches (CeT, superior and inferior mesenteric artery) which consolidate and replace these primitive arteries [7]. The RGEA is a branch of the GDA, which arises from the CeT (foregut supply), while the RCA typically arises from the SMA (midgut supply). The normal segmentation and regression of the primitive anastomoses between the foregut and midgut circulation (specifically the anastomosis between the ventral splanchnic arteries) fail in cases of arterial variation [7]. The reported aberrant RCA, therefore, likely represents a persistent, hypertrophied remnant of an early collateral channel (a primary collateral link between the CeT and SMA systems) or a direct vestige of the connection between the primitive splanchnic vessels in this region [7].

The profound variability of the right colonic vascular anatomy, often termed the “Achilles heel” of right hemicolectomy with complete mesocolic excision and central vascular ligation [8], makes this aberrant RCA origin highly significant. For oncological radicality, complete mesocolic excision requires meticulous identification and ligation of the artery at its origin [9]. In the typical medial‐to‐lateral approach, surgeons dissect toward the SMA to identify the RCA root [5, 9]. Hence, an origin from the RGEA, a vessel in the upper quadrant normally and from the CeT territory, can be easily missed or misinterpreted. Failure to correctly identify this aberrant vessel could lead to an inadequate oncological outcome by leaving behind the associated lymphovascular package. Moreover, accidental division of the RGEA without recognizing its critical role can compromise the blood supply to the stomach or duodenal stump [5]. Nevertheless, this variation also poses a direct surgical risk in gastrectomy procedures, as the RGEA is a critical vessel for stomach perfusion and mobilization [10]. Dividing the RGEA during a gastrectomy (for D2 lymphadenectomy or gastric conduit formation) without recognizing that it supplies the RCA could result in ischemia of the right colon if the marginal artery fails to rebalance the supply via the MCA. Therefore, pre‐operative vascular mapping, such as with three‐dimensional computed tomography angiography, is essential for predicting this unusual pattern and ensuring safe and complete dissection.

## Author Contributions

**Panagiotis Kokoropoulos:** visualization, writing – review and editing, supervision. **George Tsakotos:** visualization, validation, writing – review and editing. **George Triantafyllou:** conceptualization, investigation, validation, software, writing – original draft. **Orestis Lyros:** visualization, writing – review and editing, supervision. **Maria Piagkou:** supervision, writing – review and editing, methodology, investigation. **Ioannis Paschopoulos:** investigation, validation, software, writing – review and editing.

## Funding

The authors have nothing to report. The open access funding was supported by HEAL‐LINK Greece.

## Ethics Statement

The cadaver derived from the “Body Donation Program” donated to the Department of Anatomy (National and Kapodistrian University of Athens). The research was conducted ethically following the Code of Ethics of the World Medical Association (Declaration of Helsinki).

## Conflicts of Interest

The authors declare no conflicts of interest.

## Data Availability

The data that support the findings of this study are available from the corresponding author upon reasonable request.
